# Gene expression of OCT4, SOX2, KLF4 and MYC (OSKM) induced pluripotent stem cells: identification for potential mechanisms

**DOI:** 10.1186/s13000-015-0263-7

**Published:** 2015-04-24

**Authors:** Yanning Cai, Xianhua Dai, Qianhua Zhang, Zhiming Dai

**Affiliations:** School of Information Science and Technology, Sun Yat-sen University, Higher Education Mega Center, No.132 East Outer Ring Road, Guangzhou, China; SYSU-CMU Shunde International Joint Research Institute (JRI), Shunde, Guangdong China

**Keywords:** Reprogramming, Transcriptional factor, Protein-protein interaction network, Regulatory network

## Abstract

**Background:**

Somatic cells could be reprogrammed to induced pluripotent stem cells (iPS) by ectopic expression of OCT4, SOX2, KLF4 and MYC (OSKM). We aimed to gain insights into the early mechanisms underlying the induction of pluripotency.

**Methods:**

GSE28688 containing 14 gene expression profiles were downloaded from GEO, including untreated human neonatal foreskin fibroblasts (HFF1) as control, OSKM-induced HFF1 (at 24, 48, 72 h post-transduction of OSKM encoding viruses), two iPS cell lines, and two embryonic stem (ES) cell lines. Differentially expressed genes (DEGs) were screened between different cell lines and the control by Limma package in Bioconductor. KEGG pathway enrichment analysis was performed by DAVID. The STRING database was used to construct protein-protein interaction (PPI) network. Activities and regulatory networks of transcription factors (TFs) were calculated and constructed by Fast Network Component Analysis (FastNCA).

**Results:**

Compared with untreated HFF1, 117, 347, 557, 2263 and 2307 DEGs were obtained from three point post-transduction HFF1, iPS and ES cells. Meanwhile, up-regulated DEGs in first two days of HFF1 were mainly enriched in RIG-I-like receptor (RLR) and Toll-like receptor (TLR) signaling pathways. Down-regulated DEGs at 72 h were significantly enriched in focal adhesion pathway which was similar to iPS cells. Moreover, ISG15, IRF7, STAT1 and DDX58 were with higher degree in PPI networks during time series. Furthermore, the targets of six selected TFs were mainly enriched in screened DEGs.

**Conclusion:**

In this study, screened DEGs including ISG15, IRF7 and CCL5 participated in OSKM-induced pluripotency might attenuate immune response post-transduction through RLR and TLR signaling pathways.

**Virtual slides:**

The virtual slide(s) for this article can be found here: http://www.diagnosticpathology.diagnomx.eu/vs/2503890341543007.

## Background

Human embryonic stem (ES) cells have potential in cell replacement therapies using their regenerative properties. Disappointingly, there were many limitations for using of ES cells as therapeutic transplantation material, such as rejection [1], the risk of teratoma formation from residual ES cells [2] and inadequate cell number [3]. In contrast, induced pluripotent stem (iPS) cells take advantages over ES cells. It is important to highlight the need to investigate differences between iPS and ES cells. In adult tissues and organs, fully differentiated cells rarely change from one type to another. However, somatic cells can be forcibly reprogrammed to pluripotency by cell fusion, somatic cell nuclear transfer and ectopic expression of defined factors including octamer binding transcription factor 4 (OCT4), SRY related high mobility group box protein 2 (SOX2), Kruppel like factor 4 (KLF4) and myelocytomatosis viral oncogene (MYC) (known as OSKM factors) [4-6]. Takahashi and Yamanaka established a critical landmark with generation of iPS cells from fibroblasts by simple ectopic expression of OSKM factors. Notably, the 2012 Noble Prize in Physiology and Medicine was awarded to researchers for their extraordinary contribution on reprogramming somatic cells to pluripotency [7]. The advantages of OSKM-induced reprogramming to iPS cells were simplicity and robustness, as many different cell types from different species could be reprogrammed to pluripontency by ectopic expression of transcription factors [8]. Therefore, iPS cells offer an expectation for patient-specific pluripotent stem cells therapy.

Generally, many groups have shown that both human and mouse somatic cells can be reprogrammed by ectopic expression of OSKM factors to pluripotent state [9,10]. And a number of technologies were performed to understand the molecular mechanisms of cellular reprogramming mediated by OSKM factors. Gene expression profiling in fibroblasts uncovered three phases of reprogramming termed initiation, maturation and stabilization [11]. A mesenchymal-to-epithelial transition (MTE) was realized as a marker in initiation phase [12]. Also, bone morphogenic protein (BMP) signaling played a critical role in the process of OSKM-induced pluriopotency [11]. In the initiation phase, reprogrammable cells would firstly increase proliferation, then undergo histone modifications, initiate MET and followed by DNA demethylation and X-chromosome reactivation [13]. Then pluripotent genes and developmental regulators were activated which will instigate the second phase. In the last phase, the cytoskeleton was remodeled to an ESC-like state. Polo and collaborators have confirmed the initial work of three phases by further unveiling the two waves of molecular changes during reprogramming process [14]. It has been shown that the first transcriptional wave occurs in all cells and is mostly mediated by MYC, whereas the second wave is driven by OCT4/SOX2/KLF4 and is more restricted to reprogrammable cells. However, it was not fully known about how ectopic expression of OSKM induced fibroblasts to the pluripotent state.

To unravel the molecular mechanisms of this complicated process, microarray analysis was also performed to identify differentially expressed genes (DEGs) with p-value < 0.05 and enriched functions for DEGs [15]. As will be discussed during our research, we utilized this approach to analyze enriched KEGG (Kyoto Encyclopedia of Genes and Genomes) pathways and construct protein-protein interaction (PPI) network and transcriptionally regulatory network for screened DEGs.

## Methods

### Microarray data

The microarray data under the accession number GSE28688 is available at the NCBI Gene Expression Omnibus database (http://www.ncbi.nlm.nih.gov/geo/) based on the platform Illumina HumanRef-8 v3.0 expression beadchip, which composes of 14 samples including two HFF1 samples as control, six OSKM-induced HFF1 samples which were harvested 24, 48, 72 hours post-transduction, four human iPS cell lines and two human ES cell lines. Transductions were performed using pMX-based retroviral vectors each encoding the transcription factors OCT4, SOX2, KLF4, and c-MYC.

### Data preprocessing and DEGs screening

To process gene expression dataset, the log2 of expression matrix which was preprocessed by rank invariant normalization in lumi package [16] was calculated. Illumina probes were then filtered from 24526 to 17669 as different probes could map to the same gene and average expression value was set as ultimate value. DEGs were identified from different comparisons between OSKM-induced HFF1 cells and the control, between iPS cells and the control, between ES cells and the control using Limma package [17] in Bioconductor with a *t*-test under Benjamini Hochberg correction [18]. P < 0.05 and |log_2_FC| ≥ 1 were selected as the cutoff criteria.

### KEGG pathway enrichment analysis

KEGG pathway enrichment analysis for DEGs were carried out by DAVID (Database for Annotation, Visualization and Integrated Discovery) [19]. Pathways with p < 0.05 were identified as significance.

### PPI network construction

To construct PPI networks, both up- and down-regulated DEGs obtained from different comparisons were mapped to STRING [20]. The Cytoscape software was used to visualize the networks [21].

### Fast Network Component Analysis (FastNCA)

FastNCA is a fast method for determining both activities and regulatory influence for a cluster of transcription factors (TFs) [22]. To study the regulation of TFs in the complex process, six TFs [FOXF2 (forkhead box F2), GATA2 (GATA binding protein 2), FOXA3 (forkhead box A3), SMAD6 (SMAD family member 6), STAT5B (signal transducer and activator of transcription 5B) and CNTN2 (contactin 2)] whose targets were enriched in screened DEGs were chosen. Then we calculated activities of these six TFs in ES cell, iPS cell and OSKM-induced HFF1 cells and correlation between activity and gene expression of TFs using this method. To predict interactions between different TFs, STRING [20] was utilized and interaction network was visualized by Cytoscape [23]. Meanwhile, FastNCA was also performed to construct transcriptionally regulatory network for these six TFs and their target DEGs in different cell lines.

## Results

### DEGs screening

In order to gain insight into the molecular events during the early stage of reprogramming, we screened DEGs from comparisons between HFF1 cells at 24, 48, 72 h post-transduction of OSKM encoding viruses and HFF1 control, between HFF1-derived iPS cell lines and control, between the ES cell lines and control. As a result, 117, 347, 557, 2307 and 2263 DEGs were obtained, respectively (data was shown in Table 1). As shown, the number of screened DEGs in OSKM-induced HFF1 cell gradually increased with time, whilst the number of DEGs in iPS cells was nearly equal to ES cells. However, both up- and down-regulated DEGs were different in all comparisons.

**Table 1:** **Differentially expressed genes (DEGs) in different contrastive groups**

**Contrastive group**	**The number of DEGs**	**The number of up-regulated DEGs**	**The number of down-regulated DEGs**
HFF1_24 h vs HFF1	117	103	14
HFF1_48 h vs HFF1	347	234	113
HFF1_72 h vs HFF1	557	337	320
ES vs HFF1	2263	1007	1256
iPS vs HFF1	2307	699	16 8

### KEGG pathway enrichment analysis

We looked for enriched KEGG pathways of DEGs (see Table 2). Up-regulated DEGs at 24 h and 48 h post-transduction were both mainly enriched in RIG-I-like receptor (RLR) signaling pathway and Toll-like receptor (TLR) signaling pathway, especially *ISG15* (ISG15 ubiquitin-like modifier), *STAT1* (signal transducer and activator of transcription 1), *DDX58* [DEAD (Asp-Glu-Ala-Asp) box polypeptide 58], *IRF7* (interferon regulatory factor 7) and *CCL5* [chemokine (C-C motif) ligand 5]. While up-regulated DEGs at 72 h post-transduction were significantly enriched in amino acid metabolism pathway, and down-regulated DEGs were enriched in focal adhesion, regulation of actin cytoskeleton and pathway in cancer, specifically *ATCB* (actin, beta), *ITGA2* (integrin, alpha 2) and *PDGFRA* (platelet-derived growth factor receptor, alpha polypeptide). Up-regulated DEGs in comparisons between ES cells and the control, between iPS cells and the control were both mainly enriched in basal cell carcinoma and Wnt signaling pathway, especially *TP53* (tumor protein p53), but down-regulated DEGs were enriched in focal adhesion and ECM-receptor interaction pathway, especially *CCND1* (tumor protein p53) and *CD44*.

**Table 2:** **Enriched KEGG pathways in different contrastive groups**

		**KEGG pathway**	**Count**	**P value**
HFF1_24 h vs HFF1	Up-regulated gene	hsa04622: RIG-I-like receptor signaling pathway	6	9.35E-05
		hsa04620: Toll-like receptor signaling pathway	5	0.0043
		hsa04623: Cytosolic DNA-sensing pathway	4	0.0057
HFF1_48 h vs HFF1	Up-regulated gene	hsa04620: Toll-like receptor signaling pathway	6	0.0209
		hsa04622: RIG-I-like receptor signaling pathway	5	0.0249
	Down-regulated gene	hsa05410: Hypertrophic cardiomyopathy (HCM)	4	0.0479
HFF1_72 h vs HFF1	Up-regulated gene	hsa00330: Arginine and proline metabolism	6	0.0076
		hsa00480: Glutathione metabolism	5	0.0287
		hsa00250: Alanine, aspartate and glutamate metabolism	4	0.0349
	Down-regulated gene	hsa04510: Focal adhesion	13	4.57E-05
		hsa04810: Regulation of actin cytoskeleton	10	0.0056
		hsa05200: Pathways in cancer	11	0.0272
ES vs HFF1	Up-regulated gene	hsa05217: Basal cell carcinoma	14	9.58E-06
		hsa04310: Wnt signaling pathway	22	1.28E-04
		hsa05200: Pathways in cancer	36	2.29E-04
		hsa00330: Arginine and proline metabolism	10	0.0029
	Down-regulated gene	hsa04510: Focal adhesion	40	8.73E-08
		hsa00520: Amino sugar and nucleotide sugar metabolism	13	1.09E-04
		hsa04512: ECM-receptor interaction	18	2.52E-04
		hsa04142: Lysosome	20	0.0019
iPS vs HFF1	Up-regulated gene	hsa04310: Wnt signaling pathway	16	2.39E-04
		hsa05217: Basal cell carcinoma	8	0.0029
		hsa00250: Alanine, aspartate and glutamate metabolism	6	0.0041
		hsa00260: Glycine, serine and threonine metabolism	6	0.0041
	Down-regulated gene	hsa04510: Focal adhesion	56	1.46E-11
		hsa04512: ECM-receptor interaction	27	3.06E-07
		hsa00520: Amino sugar and nucleotide sugar metabolism	16	2.84E-05
		hsa04810: Regulation of actin cytoskeleton	42	1.43E-04

### PPI network construction

To identify key components of reprogramming process, we constructed PPI networks in five contrastive groups separately (data not shown). Because PPI networks were greatly complicated, the numbers of nodes and edges and proteins with higher degree in networks were shown in Tables 3 and 4.

**Table 3:** **The numbers of nodes and edges in protein-protein interaction networks of different contrasts**

	**The number of nodes**	**The number of edges**
HFF1_24 h	80	931
HFF1_48 h	217	1290
HFF1_72 h	358	1838
ES	1737	9067
iPS	1829	11765

**Table 4:** **Degree of differentially expressed proteins in protein-protein interaction network**

**HFF1_24 h**		**HFF1_48 h**		**HFF1_72 h**		**ES**		**iPS**	
**Gene**	**Degree**	**Gene**	**Degree**	**Gene**	**Degree**	**Gene**	**Degree**	**Gene**	**Degree**
ISG15	50	STAT1	59	STAT1	52	TSPO	206	TSPO	224
STAT1	50	ISG15	51	ISG15	44	TP53	168	TP53	182
DDX58	48	IFIT3	49	IRF7	41	CCND1	106	FN1	138
IFIT3	47	IRF7	49	DDX58	40	CDH1	104	IL6	136
IRF7	47	DDX58	48	TOP2A	40	MYC	96	GAPDH	131
MX1	47	IFIT1	48	MX17	39	FGF2	87	CCND1	117
RTP4	46	IFI35	47	CENPF	38	CD44	83	CDK1	117
IFIH1	45	RTP4	47	KIF2C	38	MMP9	82	CDH1	108
IFIT1	45	MX1	47	ASPM	38	COL1A2	81	CD44	103
IFI35	44	RSAD2	45	IFIT1	38	ALPL	81	RAC1	96

### TF activities calculated by FastNCA and correlation with gene expression of *TFs*

**Figure 1:**
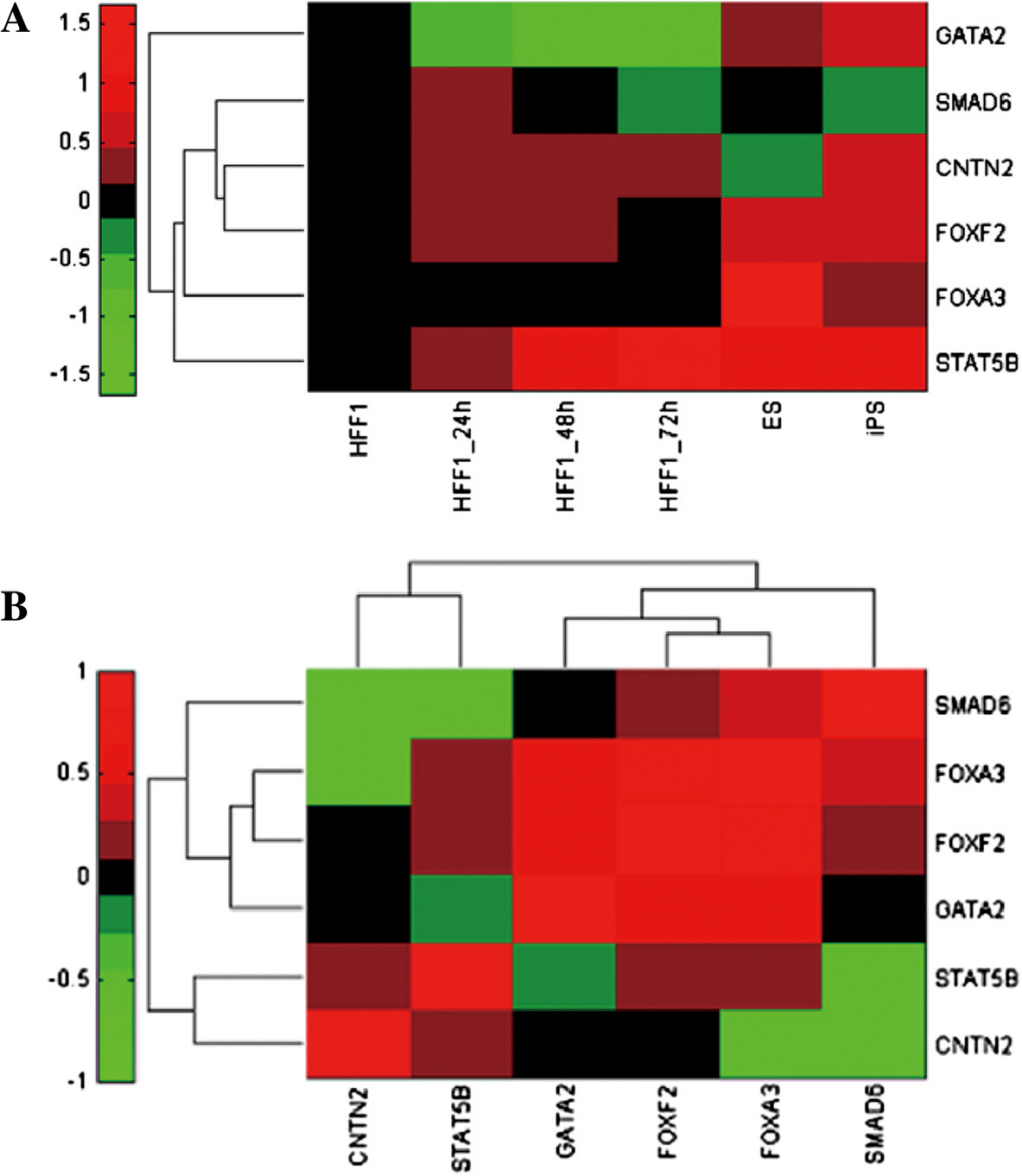
Transcription factor (TF) activities calculated by FastNCA. **A**. Predicted activities of six transcription factors (TFs) used in this study. For each TF, rows represent different cell type and columns correspond to the different TF. Black diamond represents the base level, and green diamond represents activity of TF is lower than base level. Red diamond represents activity is higher than base level. **B**. Correlation matrix between TF activities and gene expression of TF. Red diamond represents positive correlation, and green diamond represents negative correlation. Black diamond represents there is no correlation between TF activities.

Figure 1A shows the estimated activities of six TFs. HFF1 cells as control were not treated by OSKM and activities of TFs in control were set as the base level. STAT5B, FOXF2, CNTA2 and SMAD6 were activated post-transduction of OSKM encoding viruses. STAT5B retained high activity at 24, 48 h in OSKM-induced HFF1 cells, iPS cells and ES cells compared with the control. FOXF2 activity returned to base level but peaked in iPS cells and ES cells. CNTN2 activities in OSKM-induced HFF1 cells and iPS cells were higher than base level. As to SMAD6, its activity was higher than base level just at 24 h post-transduction but returned to base level at 48 h. FOXA3 activities were higher just in iPS cells and ES cells but maintained base level in OSKM-induced HFF1 cells. GATA2 activities were lower in OSKM-induced HFF1 cells but higher in iPS cells and ES cells than base level.

Figure 1B demonstrated the correlation between activities of six TFs predicted by FastNCA and gene expression of these TFs. As shown, CNTN2 and STAT5B showed strong positive correlation between activities and expression possibly due to auto- or cross-regulation. On the other hand, the activities and expression were also strongly correlated for SMAD6, FOXA3, FOXF2 and GATA2. Positive correlation stated that TFs might participate in the same biological pathway or interact between each other.

**Figure 2:**
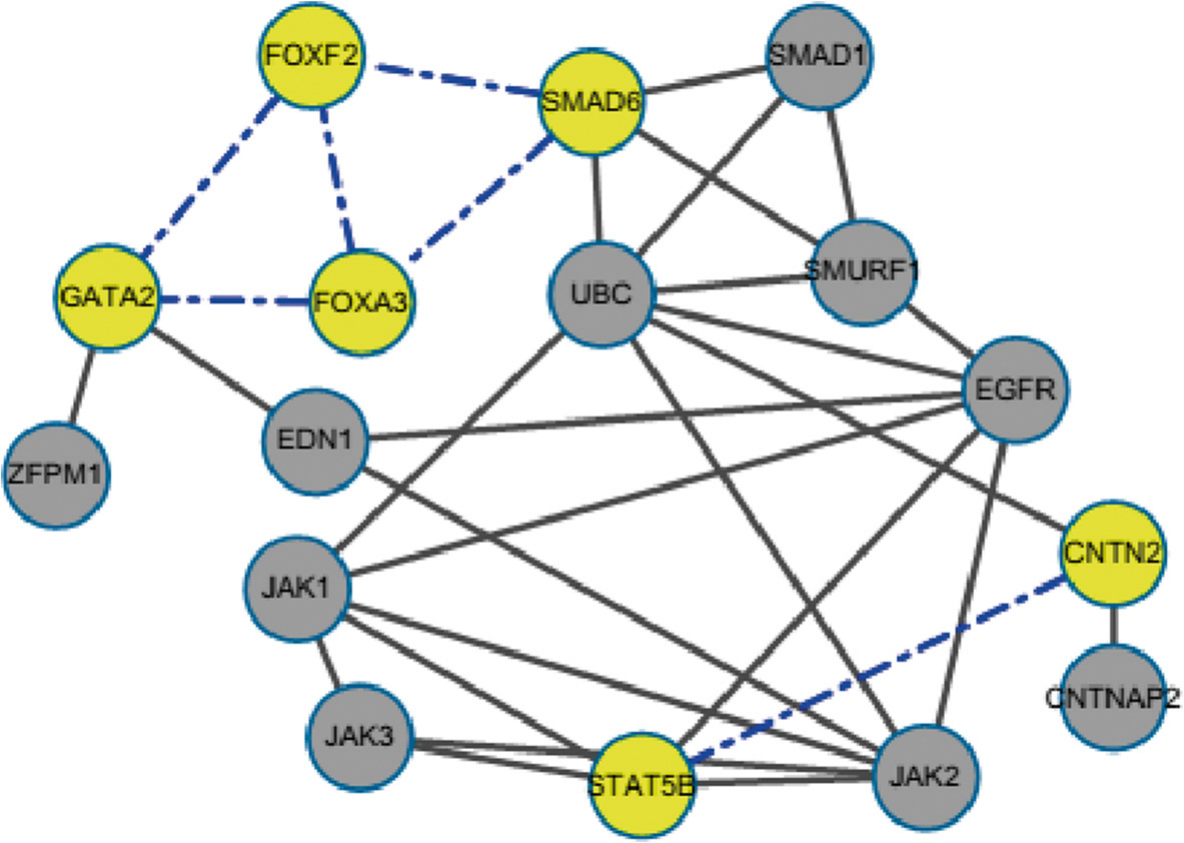
Predicted combinatorial regulation pairs of transcription factors. Yellow circle represents TFs used in this study. A black solid line indicates that the pair was supported by protein-protein interaction with STRING software. A blue dotted line indicates that the pair was predicted by FastNCA.

We wondered if predicted correlation between TF activities and gene expression could be due to the interaction of two TFs, either as a complex or otherwise. Thus, TF pairs with significant activity correlation to published protein-protein interactions were checked (Figure 2). Intriguingly, TFs which were predicted to act together showed high correlation.

### Regulatory network for TFs and DEGs

**Figure 3:**
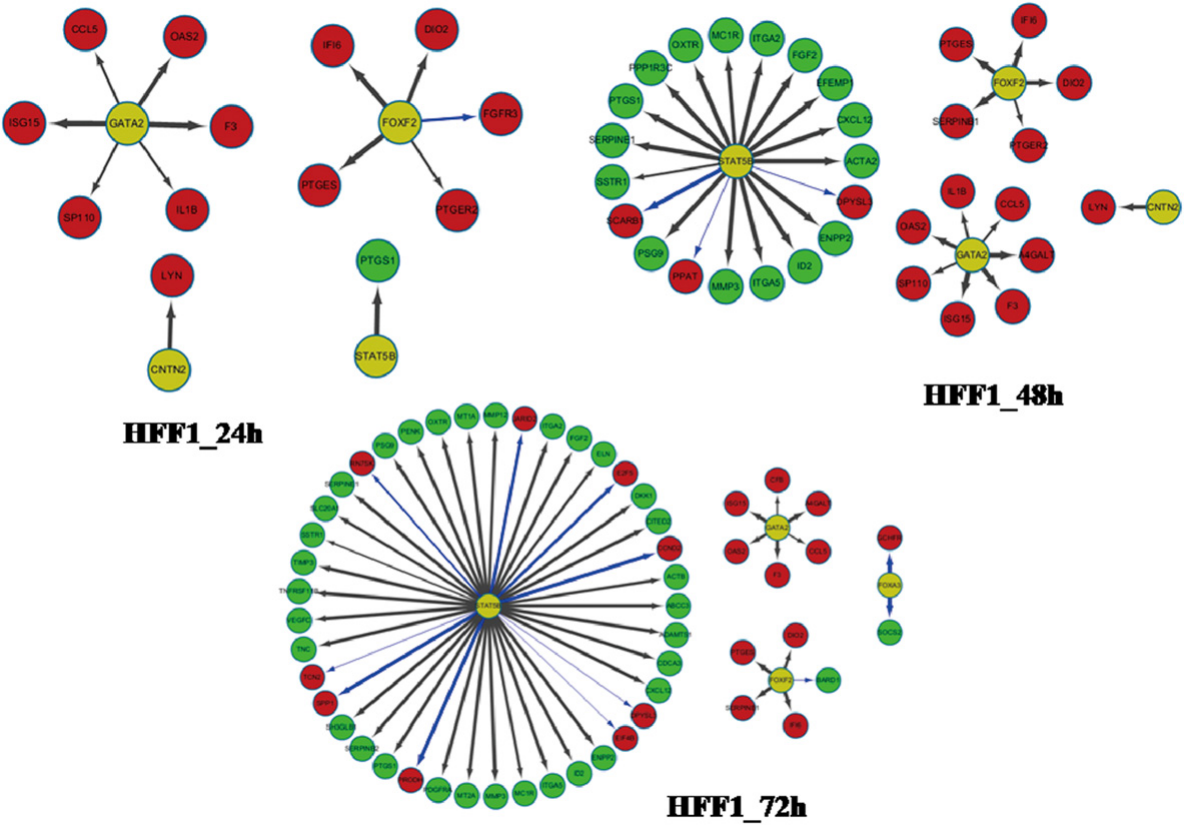
Regulatory networks for OSKM-induced HFF1. Yellow circle represents transcription factor. Red circle represents up-regulated differentially expressed gene (DEG) and green circle represents down-regulated DEG.

**Figure 4:**
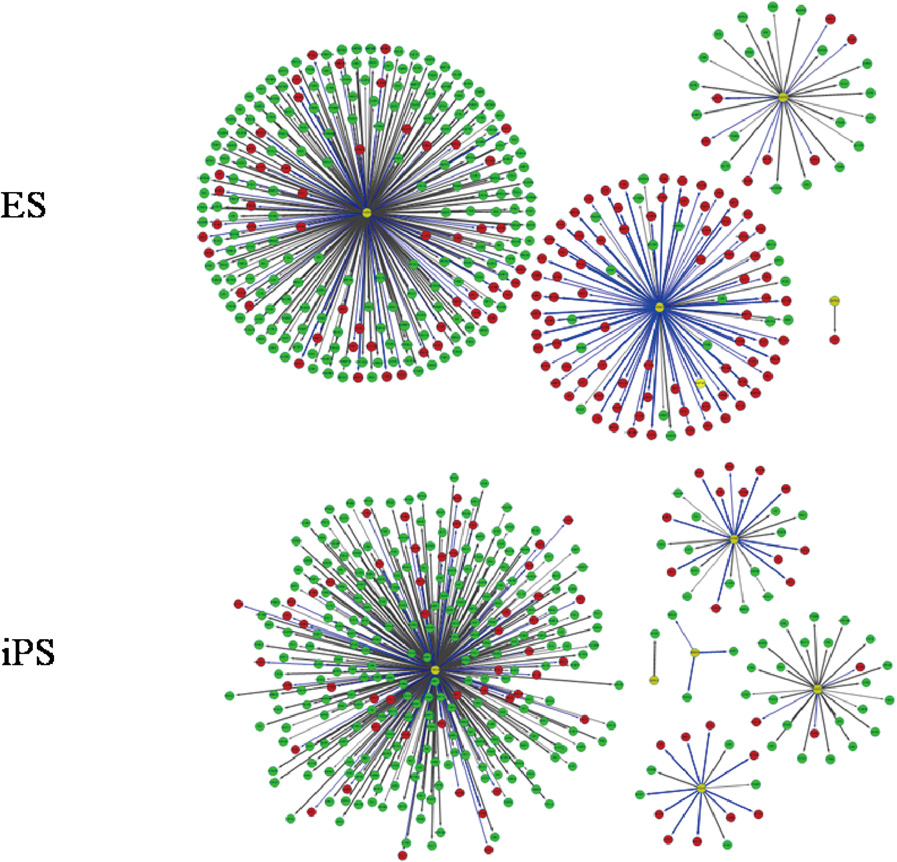
Regulatory networks for differentially expressed genes in iPS and ES cells. Yellow circle represents transcription factor. Red circle represents up-regulated differentially expressed gene (DEG) and green circle represents down-regulated DEG.

To gain insight into the enriched targets for TFs, regulatory networks were constructed for TFs and DEGs (Figures 3 and 4). In different regulatory network, the number of target DEGs varied widely (Table 5). In OSKM-induced HFF1 cells, screened DEGs were significantly targeted by STAT5B, FOXF2 and GATA2, but in iPS and ES cells, screened DEGs were mainly targeted by STAT5B, FOXA3 and FOXF2. As a result, GATA2 and FOXA3 might be the difference between somatic cells and pluripotent cells.

**Table 5:** **Degree of transcriptional factors (TF) in regulatory networks**

**TF**	**HFF1_24 h**	**HFF1_48 h**	**HFF1_72 h**	**ES**	**iPS**
CNTN2	1	2	0	2	25
STAT5B	1	19	41	245	299
FOXA3	0	0	2	98	14
FOXF2	5	5	5	28	25
GATA2	6	7	6	0	1
SMAD6	0	0	0	0	3

## Discussion

To drive somatic cells to the pluripotent state, viral transduction of OSKM factors is considered as the most robust method. Despite this, we do not fully elucidate the molecular mechanisms of reprogramming which induce somatic cells to pluripotency. To this end, we used microarray analysis to identify crucial events occurring within the first 72 hours of initiation phase. On the one hand, the screened DEGs during time series via different pathways regulated reprogramming process. On the other hand, significant TFs regulated target genes or interacted with other factors to affect reprogramming.

Following our finding from the pathway enrichment analysis, we demonstrated that up-regulated DEGs in the first 48 hours were enriched in RLR signaling pathway and TLR signaling pathway. These two pathways were reported to play an important role in immune response [24]. Although somatic cell reprogramming by viral transduction is an effective method to obtain ES-like cells, the host cell immune response acts as a roadblock to efficient reprogramming. Targeted by TFs, *ISG15*, *IRF7* and *CCL5* were significantly expressed in these two pathways. Associated with transcriptionally regulatory network, *ISG15* expression which was targeted by STAT5B and GATA2 factors was induced by virus infection. Based on accumulating evidence, it is proposed that virus-induced *ISG15* expression would conjugate ubiquitin to RIG-I to inhibit RLR signaling and attenuate immune response [25]. Together, these studies suggested that attenuation of HFF1 cell’s immune response is of benefit to reprogramming process. Meanwhile, virus infection triggers SUMOylation of IRF7 and this modification negatively regulated virus-stimulated interferon transcription [26]. And TF GATA2, targeted with *CCL5* and *ISG15*, has appeared to regulate the survival/proliferation of self-renewing stem cells [27]. In our research, up-regulated DEGs including *ISG15*, *IRF7* and *CCL5* were accordance with the aforementioned information. Importantly, the results from PPI networks in the time series showed that CCL5 interacted with MYC, IRF7, ISG15, STAT1 and DDX58 which were mostly interferon-stimulated genes [28]. Moreover, a number of reports have been published showing that MYC and other three factors induced somatic cells to pluripotent cells [29,30]. Consequently, MYC might participate in reprogramming process through interacting with CCL5 and other genes via RLR and TLR signaling pathway.

At 72 h post-transduction, down-regulated DEGs were enriched in focal adhesion and regulation of actin cytoskeleton pathways which reflected the potential establishment of cell-cell contact favorable for inducing pluripotency and were similar to iPS and ES cells, especially *ATCB* and *ITGA2. ACTB*, the target gene of STAT5B, interacted with MX1 which was a key mediator of the interferon-induced antiviral response against most of viruses through inhibiting viral primary transcription [31]. ITGA2, as a member of integrin family, could activate focal adhesion kinase and lead to cell cycle progression and cell migration which were contributed to cell reprogramming [32]. As a result, *ACTB* and *ITGA2* which were targeted by TFs played a vital role in reprogramming process likely via focal adhesion pathway.

## Conclusion

From microarray analysis for identified DEGs, results showed that gene expression of iPS cells was most similar to ES cells. Furthermore, gene expression of HFF1 cells at 72 h post-transduction was mostly alike with iPS cells. In summary, a series of interferon-stimulated genes including *ISG15*, *IRF7* might regulate cell pluripotency via RLR and TLR signaling pathways to attenuate immune response for OSKM encoding viruses, but *ATCB* and *MX1* participated in reprogramming perhaps through focal adhesion pathway. Nevertheless, future cell and animal experiments will be required to determine the role of these genes in OSKM-induced pluripotency.

## Abbreviations

ES: Human embryonic stem
iPS: Induced pluripotent stem
MTE: Mesenchymal-to-epithelial transition
BMP: Bone morphogenic protein
DEGs: Differentially expressed genes
KEGG: Kyoto Encyclopedia of Genes and Genomes
PPI: Protein-protein interaction
OCT4: Octamer binding transcription factor 4
SOX2: SRY related high mobility group box protein 2
KLF4: Kruppel like factor 4
MYC: Myelocytomatosis viral oncogene
